# High flow conditioned oxygen therapy for prevention of reintubation in critically ill patients at high risk for extubation failure: a multicenter randomised controlled trial

**DOI:** 10.1186/2197-425X-3-S1-A823

**Published:** 2015-10-01

**Authors:** G Hernandez, C Vaquero, P Gonzalez, L Colinas, S Garcia, A Canabal, A Villasclaras, R Cuena, R Fernandez

**Affiliations:** Complejo Hospitalario de Toledo, Intensive Care Medicine, Toledo, Spain; Hospital Universitario Ramon y Cajal, Intensive Care Medicine, Madrid, Spain; Hospital Universitario Infanta Sofia, Intensive Care Medicine, San Sebastian de los Reyes, Spain; Medical Council, Research Unit, Toledo, Spain; Hospital Sant Joan de Deu-Fundació Althaia, Intensive Care Medicine, Manresa, Spain; Universitat Internacional de Catalunya. CIBERES, Manresa, Spain

## Introduction

Critically ill mechanically ventilated patients are usually stratified in two levels according to the risk for reintubation. In low-risk patients high flow conditioned oxygen therapy (HFO) may reduce reintubation, whereas in high-risk patients non-invasive mechanical ventilation (NIMV) to prevent reintubation is gaining acceptance. However, data on the efficacy or safety of HFO in high-risk patients are lacking.

## Objectives

We hypothesized that HFO is an appropriate alternative to NIMV in preventing reintubation in high risk patients. The primary outcome was all-causes and respiratory-related reintubation rate within 72 hours.

## Methods

In this multicenter, randomized, noninferiority trial, we recruited 603 critically ill patients at high-risk for reintubation to be treated after extubation with either HFO (n = 290) or NIMV (n = 313). High-risk for reintubation was defined as the presence of at least any of the following criteria: >65 years, cardiac failure, moderate-to-severe COPD, APACHE II >12 points at extubation day, body mass index >30, airway patency problems, inadequate secretions management, not simple weaning, ≥2 comorbidities, and prolonged mechanical ventilation. NIMV as a rescue therapy was not allowed in the HFO group. Noninferiority was determined as a < 6% absolute difference in the risk of the primary outcome.

## Results

Respiratory-related reintubations occurred in 49 (16.9%) HFO patients and in 50 (16%) NIMV patients (risk difference 0.9%; upper limit of the unilateral 95%CI 5.9). All-causes reintubation occurred in 67 (23.1%) HFO patients and in 60 (19.2%) NIMV patients (risk difference 3.9%; upper limit of the unilateral 95%CI 9.4%).

## Conclusions

In critically ill patients at high risk, HFO was noninferior to NIMV in preventing respiratory-related reintubation, but not all-causes reintubation. The study was registered at ClinicalTrials.gov (Identifier: NCT01191489).

## References

[1] Maggiore SM, Idone FA, Vaschetto R (2014). Nasalhigh-flow versus venture mask oxygen therapy after extubation. Effects on oxygenation, comfort, and clinical outcome. Am J Respir Crit Care Med.

